# Non-Invasive Vagal Nerve Stimulation Pre-Treatment Reduces Neurological Dysfunction After Closed Head Injury in Mice

**DOI:** 10.1089/neur.2023.0058

**Published:** 2024-02-29

**Authors:** Andreia Morais, Joon Yong Chung, Limin Wu, Cenk Ayata, Bruce Simon, Michael J. Whalen

**Affiliations:** ^1^ Neurovascular Research Unit, Department of Radiology, Massachusetts General Hospital, Harvard Medical School, Charlestown, Massachusetts, USA.; ^2^ Department of Pediatrics, Massachusetts General Hospital, Harvard Medical School, Charlestown, Massachusetts, USA.; ^3^ ElectroCore, Inc., Basking Ridge, New Jersey, USA.

**Keywords:** closed head injury, inflammation, vagus nerve stimulation

## Abstract

Non-invasive vagus nerve stimulation (nVNS) has recently been suggested as a potential therapy for traumatic brain injury (TBI). We previously demonstrated that nVNS inhibits cortical spreading depolarization, the electrophysiological event underlying migraine aura, and is relevant to TBI. Our past work also suggests a role for interleukin-1 beta (IL-1β) in cognitive deficits after closed head injury (CHI) in mice. We show that nVNS pre-treatment suppresses CHI-associated spatial learning and memory impairment and prevents IL-1β activation in injured neurons, but not endothelial cells. In contrast, nVNS administered 10 min after CHI was ineffective. These data suggest that nVNS prophylaxis might ameliorate neuronal dysfunction associated with CHI in populations at high risk for concussive TBI.

## Introduction

Traumatic brain injury (TBI) is an important public health problem in the United States and worldwide. The estimated 5.3 million Americans living with TBI-related disabilities face numerous challenges in returning to a full and productive life.^1^ The long-term cognitive, psychosocial, and physical deficits after TBI lead to an economic burden estimated at $60 billion annually.^2,3^

Diffuse or concussive TBI caused by vehicular accidents, sports concussions, and explosive devices is associated with cognitive and psychiatric sequelae that may cause significant functional impairment through unknown mechanisms.^4^ Inflammation has a significant role in aggravating secondary injury after human and experimental TBI.^5^ In particular, we previously showed that closed head injury (CHI) induced endothelial cell activation of interleukin-1 beta (IL-1β), and global deficiency of interleukin-1 receptor 1 (*IL-1R1^–/–^*) prevented post-injury cognitive deficits in mice.^6^

The vagus nerve is the major visceral efferent (parasympathetic) output and carries visceral sensory afferents to the brainstem. Indeed, afferents constitute 80% of all nerve fibers in the vagus.^7^ Both efferent and afferent directions are linked to anti-inflammatory pathways, the efferent through alpha-7 nicotinic acetylcholine receptors on immune cells and the afferent by the nucleus tractus solitarius.^8^ Vagus nerve stimulation (VNS) is U.S. Food and Drug Administration approved for treating epilepsy, major depression, migraine, and cluster headache.^9^ Invasive VNS has been shown to reduce histopathology and improve functional outcomes in experimental models of cerebral contusion and blast injury.^10–24^ However, VNS (invasive or non-invasive) has not been reported in a model of mild TBI or concussion. Here, we evaluated whether non-invasive VNS (nVNS) prevents cognitive dysfunction after closed-head TBI and whether neuronal and endothelial inflammation plays a role.

## Methods

### Ethics

All procedures were performed with approval from the Massachusetts General Hospital Institutional Animal Care and Use Committee in accordance with the NIH Guide for Care and Use of Laboratory Animals. Studies were performed according to the Animal Research: Reporting of In Vivo Experiments (ARRIVE) guidelines, and all data were obtained by investigators blinded to study groups.

### Animals

A total of 112 C57Bl/6J (3-month-old) male mice (25–35 g; obtained from The Jackson Laboratory, Bare Harbor, ME) were used in the study. Mice were acclimated in the animal holding facility (thermostatic control at 22°C ± 1°C, 40–70% humidity, and 12-h light/dark cycle) for at least 3 days before experiments and allowed access to a standard rodent diet and water *ad libitum*. Mice were separated into two cohorts: nVNS pre- and post-treatment. Each cohort had four experimental groups: sham-nFNS (non-invasive femoral nerve stimulation; *N* = 16/10, pre/post); sham-nVNS (*N* = 16/10, pre/post); CHI-nFNS (*N* = 12/10, pre/post); and CHI-nVNS (*N* = 12/10, pre/post). An additional 32 sham-nFNS (*N* = 3), sham-nVNS (*N* = 3), CHI-nFNS (*N* = 5), and CHI-nVNS (*N* = 5) animals were used in the western blot analyses for only the pre-treatment groups.

### Non-invasive vagus nerve stimulation paradigms

**FIG. 1.**
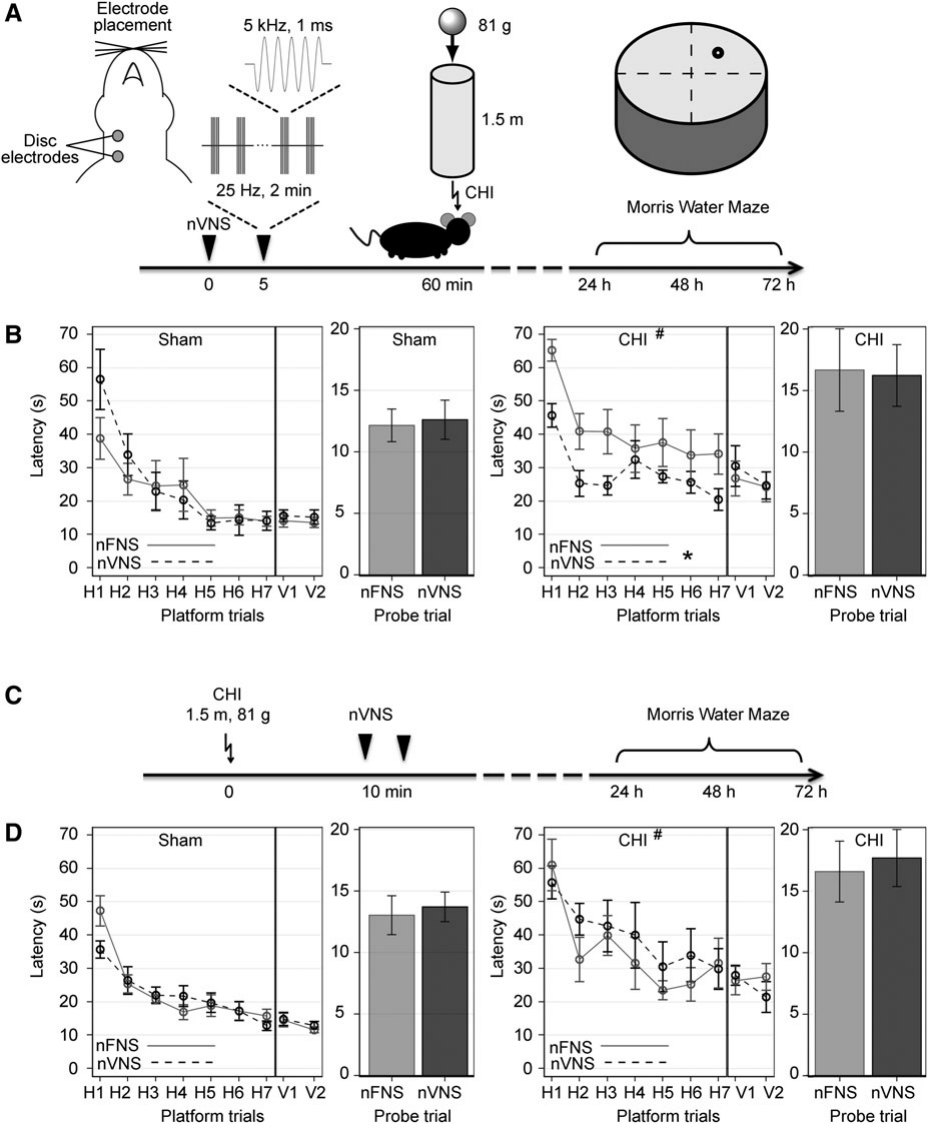
Neurological dysfunction after CHI and nVNS evaluated by MWM. (**A**) nVNS pre-treatment timeline (*n* = 12 CHI and 16 sham per group). (**B**) Compared to sham injury, CHI increased latency to the hidden and visible platforms (^#^vs. sham, *p* < 0.001 for group, two-way RM ANOVA). nVNS pre-treatment ameliorated the CHI effect in hidden (*nFNS, *p* < 0.05, two-way RM ANOVA), but not visible, platform trials. There was no difference in the probe trial among the groups. (**C**) nVNS post-treatment timeline (*n* = 10 per group). (**D**) Compared to sham, CHI increased latency to hidden and visible platform trials (^#^*p* < 0.01, two-way RM ANOVA), but nVNS presented similar latencies to nFNS in both CHI and sham-injured groups. Probe trial performance did not differ among the experimental groups. CHI, closed head injury; MWM, Morris water maze; nFNS, non-invasive femoral nerve stimulation; nVNS, non-invasive vagus nerve stimulation; RM ANOVA, repeated-measures analysis of variance.

A custom-made gammaCore non-invasive nVNS device (electroCore LLC, Basking Ridge, NJ) was used to deliver the stimulation (a 5-kHz sine wave for 1 ms repeating at a rate of 25 Hz) by placing two disk electrodes (2 mm in diameter, 3 mm separation) on the shaved skin overlying the left or right vagal nerves (2–4 mm lateral to midline from the larynx) with conductive gel (Fig. 1A). nVNS was delivered on the right and left for 2 min each, with a 5-min rest in between. Control mice received an identical two electrical stimulations sequentially on the right and left anterior thigh area overlying the quadriceps femoris with a 5-min interval in between (nFNS). The pre-treatment cohort received nVNS or nFNS 60 min before sham or CHI (Fig. 1A). The post-treatment cohort received nVNS or nFNS 10 min after sham or CHI (Fig. 1C).

### Closed head injury

The CHI model was used as previously described,^6^ with minor modifications. Mice were anesthetized with 2.5% isoflurane (Anaquest, Memphis, TN) for 90 sec in 70% N_2_O/30% O_2_ using a Fluotec 3 vaporizer (Colonial Medical, Amherst, NH). Mice were placed on a Kimwipe napkin (Kimberly-Clark, Irving, TX) and grasped by the tail, and the head was positioned under a hollow tube (diameter, 10 mm). A metal bolt (81 g) was dropped 1.5 m onto the dorsal aspect of the skull between the coronal and lambdoid sutures. The head readily penetrated the Kimwipe after impact in the anterior-posterior plane. Sham-injured mice were subjected to anesthesia without a weight drop.

### Morris water maze

The Morris water maze (MWM) was performed as previously described,^6^ with minor modifications. Each mouse was subjected to seven hidden platform trials (90 sec maximum, two or three daily trials). Probe trials were performed 24 h after the last hidden platform trial by allowing mice to swim in the tank for 30 sec and recording the time spent in the target quadrant. For visible platform trials, the platform location remained the same position as the hidden trials, but was made plainly visible above the water level. Mice were subjected to two visible platform trials.

### Isolation of brain endothelium and neurons

Mice were transcardially perfused with phosphate-buffered saline (PBS), and the brain was digested using a neural tissue dissociation kit (Roche Diagnostic GmbH, Penzberg, Germany) and mechanically dissociated with a plastic pipette. After centrifugation at 1000*g* (7 min), the cell pellet was resuspended and incubated with myelin removal beads (Miltenyi Biotec Inc., Charlestown, MA) for 40 min on ice. After washing in PBS, Dynabeads (ThermoFisherScientific, Waltham, MA) conjugated to anti-CD31 (#550274; BD Pharmingen, San Diego, CA) were added and a magnetic separator was used to recover the bead-bound cells. Neurons were isolated by negative selection. Isolated cells were frozen at −80°C.

### Western blot

Isolated microvessels and neurons were homogenized in radioimmunoprecipitation assay buffer (EMD Millipore, Burlington, MA) with phosphatase and protease inhibitors (ThermoFisherScientific) and subjected to western blotting as previously described.^6^ Membranes were incubated overnight at 4°C with the following primary antibodies: anti-β-actin antibody (1:10,000; #5125; Cell Signaling Technology, Danvers, MA); anti-IL-1β antibody (1:1000; #AB9722; Abcam, Cambridge, MA). After incubation with peroxide-conjugated secondary antibodies (1:2000; #7074; Cell signaling Technology), visualization was enhanced by electrochemiluminescence (EMD Millipore) detection. The results were normalized to β-actin. Optical density was assessed using ImageJ software (NIH, Bethesda, MD).

### Statistical analysis

Mice were randomly assigned to injury and treatment groups. Sample sizes were calculated using GPower (version 3.1) to detect an effect size of 50% and achieve 80% power (α = 0.05). No animal was excluded. Researchers performing the CHI and MWM were blinded to the treatment arm. Data were analyzed by a separate researcher (M.J.W.) blinded to the study arms. Two-way repeated-measures (RM) analysis of variance (ANOVA) was used to analyze the MWM data. Inflammation data were analyzed by either two-way ANOVA or non-parametric *t*-test; the test is identified in the results. Data are expressed as mean ± standard error of the mean. Statistical analysis and graphs were made in SAS Studio (version 9.4; SAS Institute Inc., Cary, NC). *p* < 0.05 was considered significant.

## Results

### Non-invasive vagus nerve stimulation pre-treatment improves Morris water maze performance after closed head injury

In the first cohort, a group of mice had their vagus or femoral nerve non-invasively stimulated 60 min before the CHI (Fig. 1A). In contrast, another group underwent the same procedure without the injury. CHI mice presented higher latency in finding the hidden and visual (*p* < 0.001, two-way RM ANOVA) platforms (Fig. 1B). Interestingly, nVNS improved the spatial memory task by decreasing the latency for the mouse to reach the hidden (*p* < 0.05, two-way RM ANOVA), but not the visual, platform (Fig. 1B). Neither CHI nor nVNS had interference with the trial performance. The differences in the distance described were not attributable to the swimming speed; all groups presented similar swimming performances along the MWM testing days (Table 1). In the second cohort, nVNS was performed 10 min after CHI (Fig. 1C). Similar to the first cohort, injured mice had more difficulties in performing the task, judged by the higher latencies to find both hidden (*p* < 0.01, two-way RM ANOVA) and visual platforms (*p* < 0.0001, two-way RM ANOVA), despite the location of the stimulation (nFNS or nVNS; Fig. 1D). Swimming speed did not alter among the injury or stimulated groups (Tabel 1).

**Table 1.** Averaged Swimming Speed Throughout MWM Trials

***nVNS pre-treatment***				
***Trials***	***CHI-nFNS***	***CHI-nVNS***	***Sham-nFNS***	***Sham-nVNS***
H1	14 ± 2	13 ± 2	15 ± 1	14 ± 2
H2	12 ± 3	12 ± 2	12 ± 2	13 ± 2
H3	11 ± 2	12 ± 2	12 ± 3	12 ± 2
H4	10 ± 3	10 ± 2	11 ± 2	11 ± 2
H5	11 ± 3	11 ± 2	11 ± 2	10 ± 2
H6	10 ± 3	8 ± 2	9 ± 2	9 ± 2
H7	10 ± 3	7 ± 2	9 ± 3	8 ± 2
Probe	17 ± 4	15 ± 3	16 ± 3	16 ± 2
V1	11 ± 3	11 ± 3	9 ± 2	9 ± 2
V2	11 ± 3	10 ± 3	10 ± 2	11 ± 2

***nVNS post-treatment***				
***Trials***	***CHI-nFNS***	***CHI-nVNS***	***Sham-nFNS***	***Sham-nVNS***
H1	15 ± 3	13 ± 2	14 ± 2	14 ± 2
H2	12 ± 2	12 ± 1	12 ± 2	11 ± 2
H3	13 ± 3	11 ± 3	11 ± 3	11 ± 3
H4	10 ± 3	9 ± 2	10 ± 2	10 ± 3
H5	12 ± 3	10 ± 2	9 ± 2	10 ± 3
H6	11 ± 2	10 ± 3	9 ± 2	9 ± 2
H7	10 ± 2	9 ± 2	8 ± 2	8 ± 1
Probe	18 ± 3	17 ± 2	17 ± 3	16 ± 4
V1	11 ± 2	12 ± 3	11 ± 5	10 ± 2
V2	10 ± 2	9 ± 2	10 ± 2	8 ± 3

There was no difference among the groups.

MWM, Morris water maze; CHI, closed head injury; nFNS, non-invasive femoral nerve stimulation; nVNS, non-invasive vagus nerve stimulation.

### Pre-injury non-invasive vagus nerve stimulation reduces neuronal interleukin-1β activation after closed head injury

**FIG. 2.**
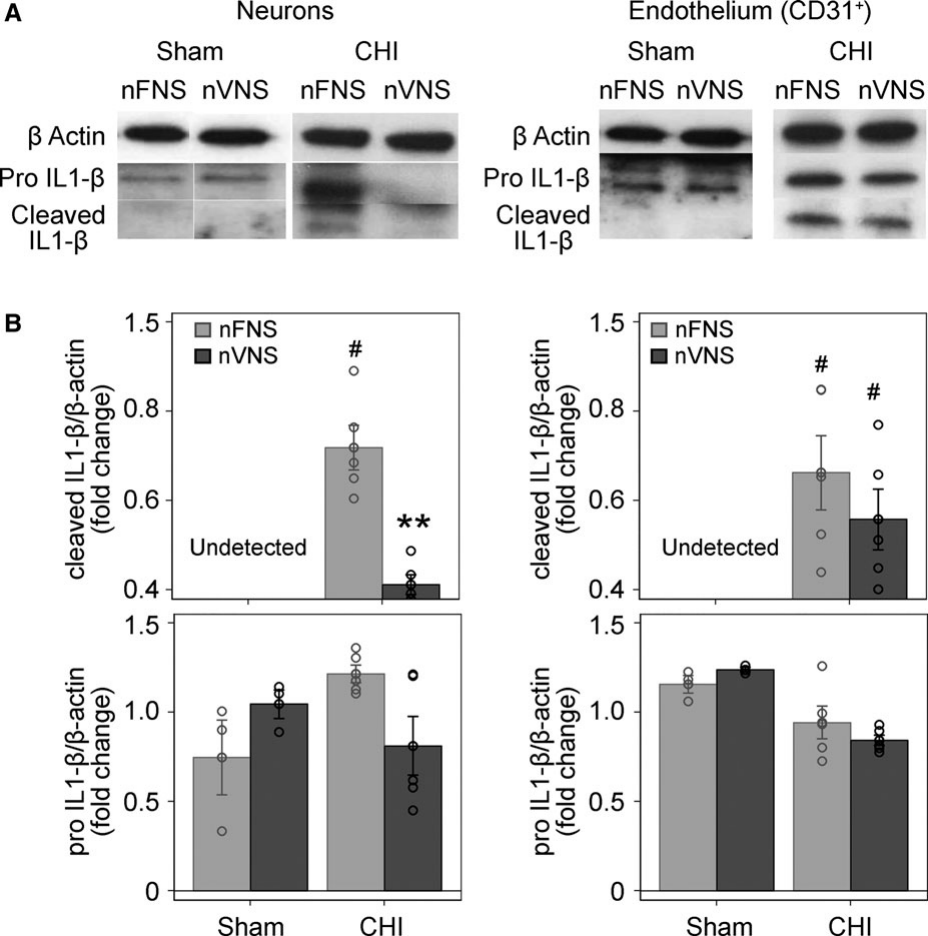
Qualitative (**A**) and quantitative (**B**) effect of nVNS on IL-1b activation after CHI in neurons and CD31^+^ endothelial cells. Cleaved IL-1b was not detected in shams, but was robustly induced by CHI (^#^*p* < 0.01, two-way ANOVA) in neurons and endothelium. In neurons, nVNS significantly prevented CHI-associated upregulation of cleaved IL-1b expression (**p < 0.01, two-way ANOVA), but had no impact over endothelial cleaved IL-1b. Pro-IL-1b expression was upregulated in neurons in CHI-nFNS compared to sham-nFVS (^#^*p* < 0.05, non-parametric *t*-test) in brain endothelium and was not modulated by nVNS. *N* = 3 sham and 5 CHI/group. ANOVA, analysis of variance; CHI, closed head injury; IL, interleukin; nFNS, non-invasive femoral nerve stimulation; nVNS, non-invasive vagus nerve stimulation.

We previously reported that neuronal IL1-β activation is associated with post-injury cognitive deficits after a single CHI in adult mice and that IL-1R1 deletion prevented these deficits.^6^ Figure 2 shows the results of western blots for IL-1β in neurons and endothelium (CD31^+^) isolated by immunopanning at 24 h after sham or CHI. Cleaved (activated) IL-1β (17 kDa) was not detected in sham-injured mice, but could be observed in the CHI brain in both neurons and endothelium. Pro-IL-1β (32 kDa) could be detected in CHI and sham-injured animals in higher concentrations in neurons and endothelium (*p* < 0.05, sham-nFNS vs. sham-nVNS, non-parametric *t*-test). Most important, nVNS prevented the injury-related increase of cleaved (*p* < 0.01, two-way ANOVA), specifically in neurons.

## Discussion

CHI models with free head rotation/acceleration, such as the one described herein,^6^ have been developed to model sports concussions, which feature functional disturbances without overt brain damage assessed by conventional imaging techniques. Here, we show that nVNS applied before, but not after, CHI attenuates spatial memory impairment and reduces neuronal IL1-β maturation associated with cognitive deficits in this model. VNS has been proposed to improve cognition in healthy volunteers, persons with epilepsy, and non-demented persons with Alzheimer's disease (AD).^25^ To our knowledge, this is the first demonstration of the beneficial effects of nVNS in a concussion model.

We previously reported that IL-1β/IL-1R1 signaling contributes to cognitive deficits post-CHI and that genetic deletion of IL-1R1 improved cognitive outcomes post-CHI.^6^ Given that nVNS may reduce IL-1β activation,^26–29^ it became a strong candidate for therapies treating/preventing secondary sequelae of CHI. Indeed, nVNS pre-treatment was efficacious in preventing CHI-induced behavior impairment. However, the mechanisms by which nVNS attenuates the MWM deficits caused by CHI are still unclear. It has been reported that nVNS modulates inflammation, edema, and blood–brain barrier disruption in cerebral contusion models.^13,14,16,23^ Here, we show that nVNS reduces CHI-induced IL-1β maturation in neurons, but not in endothelial cells, suggesting a direct effect of nVNS on the central and peripheral nervous systems interconnected with vagal nerve afferents.

It is well accepted that neuroinflammation is responsible for beneficial and detrimental effects post-TBI, facilitating repair and contributing to secondary brain injury.^5^ It is interesting to note the specificity of the nVNS anti-inflammatory effect in neurons. In a fluid percussion injury model, nVNS specifically prevented the loss of GABA neurons within the cerebral cortex and, possibly, hippocampal formation.^17^ Therefore, the neuronal specificity observed in the pre-CHI nVNS group on inflammation in the current study may be associated with central anti-inflammatory pathways rather than systemic cholinergic modulation.

Using the cortical spreading depolarization model relevant to TBI pathophysiology,^30^ we have previously reported that nVNS is effective for migraine prophylaxis and treatment.^31^ In the current study, post-treatment nVNS had no efficacy on CHI outcome. However, nVNS 1 h pre-treatment was efficacious in preventing behavioral impairment and neuronal inflammation. Centrally, nVNS increases the release of norepinephrine and serotonin.^32,33^ This is the primary mechanism by which nVNS affects epilepsy, major depression, and migraine.^34–36^ However, the monoamine effects on TBI remain unclear.^37^ Interestingly, a case-control study found that patients with post-traumatic epilepsy achieved better outcomes after nVNS therapy than persons with other epilepsy etiologies.^38^ Therefore, nVNS probably acts on TBI by additional mechanisms other than the ones known for epilepsy treatment.

A possible mechanism by which nVNS reduces IL1-β specifically in neurons could be its action on inflammasome activation.^23^ We previously reported that inflammasome activity contributes to cognitive dysfunction in CHI models.^6,39^ A growing body of evidence suggests that neuronal NLRP1 (nucleotide-binding domain-like receptor protein 1) inflammasome activity contributes to AD,^40–43^ hence further studies evaluating the nVNS role on neuronal NLRP1 inflammasome are needed. It is important to highlight that the current study is the first to document a therapy that has specificity to neuronal inflammation in a TBI model, which is interesting given that increased Il-1β impairs synaptic transmission.^44^

Previous studies have shown that invasive VNS beginning 2 or 24 h after fluid percussion injury improves post-injury MWM performance.^19,20^ The researchers posited that changes in norepinephrine signaling might underlie the protective effects of VNS.^19,20^ Notably, in our study, the 10-min post-injury stimulation group was not protected from MWM deficits. One possibility might be that neuronal IL-1β is responsible for the post-injury cognitive deficits, and nVNS administered post-CHI did not reduce neuronal IL-1β beta activation, rendering nVNS ineffective. We did not assess this possibility with western blot analysis for a number of reasons. First, we do not know whether neuronal IL-1β activation is necessary or sufficient to produce post-injury MWM deficits. To determine this, we would need reagents to specifically inhibit neuronal IL-1β production either before or immediately after CHI. A floxed IL-1β mouse has recently been generated,^45^ and future studies using floxed IL-1β with an inducible neuronal Cre system could be used to answer this question by eliminating IL-1β from neurons, but not endothelium or other cell types.

Experiments using a neuronal IL-1R1 KO mouse would not answer the question because active IL-1β is made by endothelium as well as neurons; absence of the neuronal IL-1β receptor would not allow us to distinguish between the importance of neuronal versus endothelial IL-1β in post-injury cognitive deficits. Second, it is possible that IL-1β signaling in a subpopulation of neurons (e.g., dentate gyrus) may be critical for post-injury cognitive deficits. If so, western blot on neurons isolated from the entire brain would lack sensitivity to detect IL-1β effects; immunohistochemistry could be used, but the IL-1β antibodies do not distinguish between full-length and activated forms. Third, it is possible that mechanisms other than or in addition to IL-1β may be critical and the reduction in IL-1β by nVNS at any time point may be an epiphenomenon. Hence, we hypothesize that neuronal IL-1β may be involved in the protective mechanism of nVNS with regard to post-injury cognitive function, but proof of this hypothesis requires further studies beyond the scope of the current study.

Here, most important, a single non-invasive administration of VNS, sequentially to each vagus nerve, before CHI, effectively reduces MWM deficits in a clinically relevant concussion model. VNS has been paired with motor training to enhance rehabilitation after stroke,^46^ and timing of VNS was critical in a post-traumatic stress disorder model.^47^ Further studies are needed to optimize the dose and timing of nVNS in CHI models, the durability of the effects of nVNS, and evaluate other mechanisms of protection, including adrenergic effects. With the advent of commercially available nVNS devices by prescription, the current study provides evidence that nVNS could be used as a prophylaxis of cognitive sequelae of concussion in athletes, military personnel, and others at risk for TBIs.

## Abbreviations Used

AD: Alzheimer's disease
ANOVA: analysis of variance
CHI: closed head injury
IL-1β: interleukin-1 beta
*IL-1R1^–/–^*: interleukin-1 receptor 1
MWM: Morris water maze
nFNS: non-invasive femoral nerve stimulation
NLRP1: nucleotide-binding domain-like receptor protein 1
nVNS: non-invasive vagus nerve stimulation
PBS: phosphate-buffered saline
RM ANOVA: repeated-measures ANOVA
TBI: traumatic brain injury
VNS: vagus nerve stimulation
